# Regulatory T cell therapy is associated with distinct immune regulatory lymphocytic infiltrates in kidney transplants

**DOI:** 10.1016/j.medj.2024.11.014

**Published:** 2024-12-27

**Authors:** Oliver McCallion, Amy R. Cross, Matthew O. Brook, Conor Hennessy, Ricardo Ferreira, Dominik Trzupek, William R. Mulley, Sandeep Kumar, Maria Soares, Ian S. Roberts, Peter J. Friend, Giovanna Lombardi, Kathryn J. Wood, Paul N. Harden, Joanna Hester, Fadi Issa

**Affiliations:** 1 Nuffield Department of Surgical Sciences, University of Oxford, Oxford OX3 9DU, UK; 2 Department of Renal Medicine, Oxford University Hospitals NHS Foundation Trust, Oxford OX3 7LH, UK; 3 Centre for Human Genetics, Nuffield Department of Medicine, University of Oxford, Oxford OX3 7BN, UK; 4 Department of Nephrology, Monash Medical Centre & Department of Medicine, Monash University, Clayton, VIC 3168, Australia; 5 Advanced Therapy Manufacturing (GMP) Unit, Guy’s & St Thomas’ NHS Foundation Trust and King’s College London, London SE1 9RT, UK; 6 Department of Cellular Pathology, Oxford University Hospitals NHS Foundation Trust, Oxford OX3 9DU, UK; 7 MRC Centre for Transplantation, King’s College London, London SE1 9RT, UK

## Abstract

**Background:**

Adoptive transfer of autologous regulatory T cells (Tregs) is a promising therapeutic strategy aimed at enabling immunosuppression minimization following kidney transplantation. In our phase 1 clinical trial of Treg therapy in living donor renal transplantation, the ONE Study (ClinicalTrials.gov: NCT02129881), we observed focal lymphocytic infiltrates in protocol kidney transplant biopsies that are not regularly seen in biopsies of patients receiving standard immunosuppression.

**Methods:**

We present 7 years of follow-up data on patients treated with adoptive Treg therapy early post-transplantation who exhibited focal lymphocytic infiltrates on a 9-month protocol biopsy. We phenotyped their adoptively transferred and peripherally circulating Treg compartments using CITE-seq and investigated the focal lymphocytic infiltrates with spatial proteomic and transcriptomic technologies.

**Findings:**

Graft survival rates were not significantly different between Treg-treated patients and the control reference group. None of the Treg-treated patients experienced clinical rejection episodes or developed *de novo* donor-specific antibodies, and three of ten successfully reduced their immunosuppression to tacrolimus monotherapy. All Treg-treated patients who underwent a protocol biopsy 9 months post-transplantation exhibited focal lymphocytic infiltrates. Spatial profiling analysis revealed prominent CD20^+^ B cell and regulatory (*IKZF2, IL10, PD-L1, TIGIT*) signatures within cell-therapy-associated immune infiltrates, distinct from the pro-inflammatory myeloid signature associated with rejection biopsies.

**Conclusions:**

We demonstrate for the first time that immune cell infiltrates in transplanted kidneys can occur following adoptive Treg therapy in humans, potentially facilitating within-graft T:B cell interactions that promote local immune regulation.

**Funding:**

This work was funded by the 7^th^ EU Framework Programme, grant/award no. 260687, and the National Institute for Health Research (NIHR).

## Graphical abstract

**Graphical abstract:**
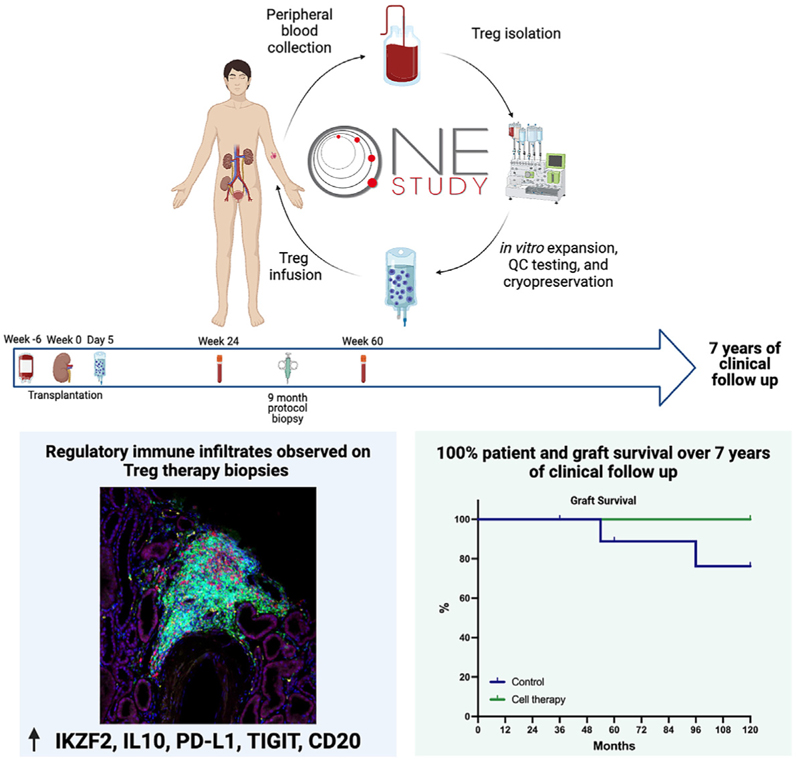

## Introduction

Kidney transplantation remains the treatment of choice for chronic kidney disease (CKD) or end-stage renal disease (ESRD). Despite advances in transplantation and immunosuppressive regimens, kidney allograft recipients still rely on potent immunosuppression to prevent graft rejection. These pharmacological agents are associated with significant side effects, including increased lifetime risk of malignancy, infections, cardiovascular disease, and nephrotoxicity.^1–5^ These challenges have sparked interest in methods to reduce immunosuppressive burden, with regulatory T cell (Treg)-based therapeutics at the forefront.^6–8^

Tregs are characterized by the cell surface expression of CD25 and CD4 and the transcription factor FOXP3^9^ and act through multiple non-redundant mechanisms to modulate dendritic cell activity as well as T cell activation, maturation, and effector function.^10,11^ For example, cell surface expression of CTLA4 prevents the action of antigen-presenting cells (APCs) and the activation of effector T cells,^12–14^ while production of immunomodulatory cytokines such as interleukin (IL)-10 and transforming growth factor β (TGF-β) and surface expression of the ectonucleotidases CD39 and CD73 can constrain immune activation in local niches.^15^ In addition to their systemic immune regulatory effects, modulation of local immune responses within the tissue is also likely to be important.^16,17^

In the phase 1 ONE Study (ClinicalTrials.gov: NCT02129881), we demonstrated that Treg therapy in kidney transplant recipients is safe, feasible, and potentially efficacious.^18,19^ The intravenous administration of 1–10 3 10^6^/kg autologous *ex*-*vivo-*expanded Tregs 5 days post-transplantation and in the absence of induction immunosuppression enabled safe weaning to tacrolimus monotherapy in a third of patients, together with significantly reduced infection events. However, protocol-driven biopsies at 9 months post-transplantation revealed significant lymphocytic graft infiltrates resembling rejection, which confounded clinical decision-making.^19^ Assessing the nature of these histological changes is crucial to understanding the impact and likely safety of Treg ther-apy. Here, we report 7-year outcome data from the ONE Study and employ advanced spatial profiling techniques on the biopsies. We uncover interactions between T and B lymphocytes within the transplant, presenting new insights into the local signatures of immune regulation following Treg therapy.

## Results

### Patients treated with autologous Treg therapy exhibit long-term stable graft function

**Figure 1:**
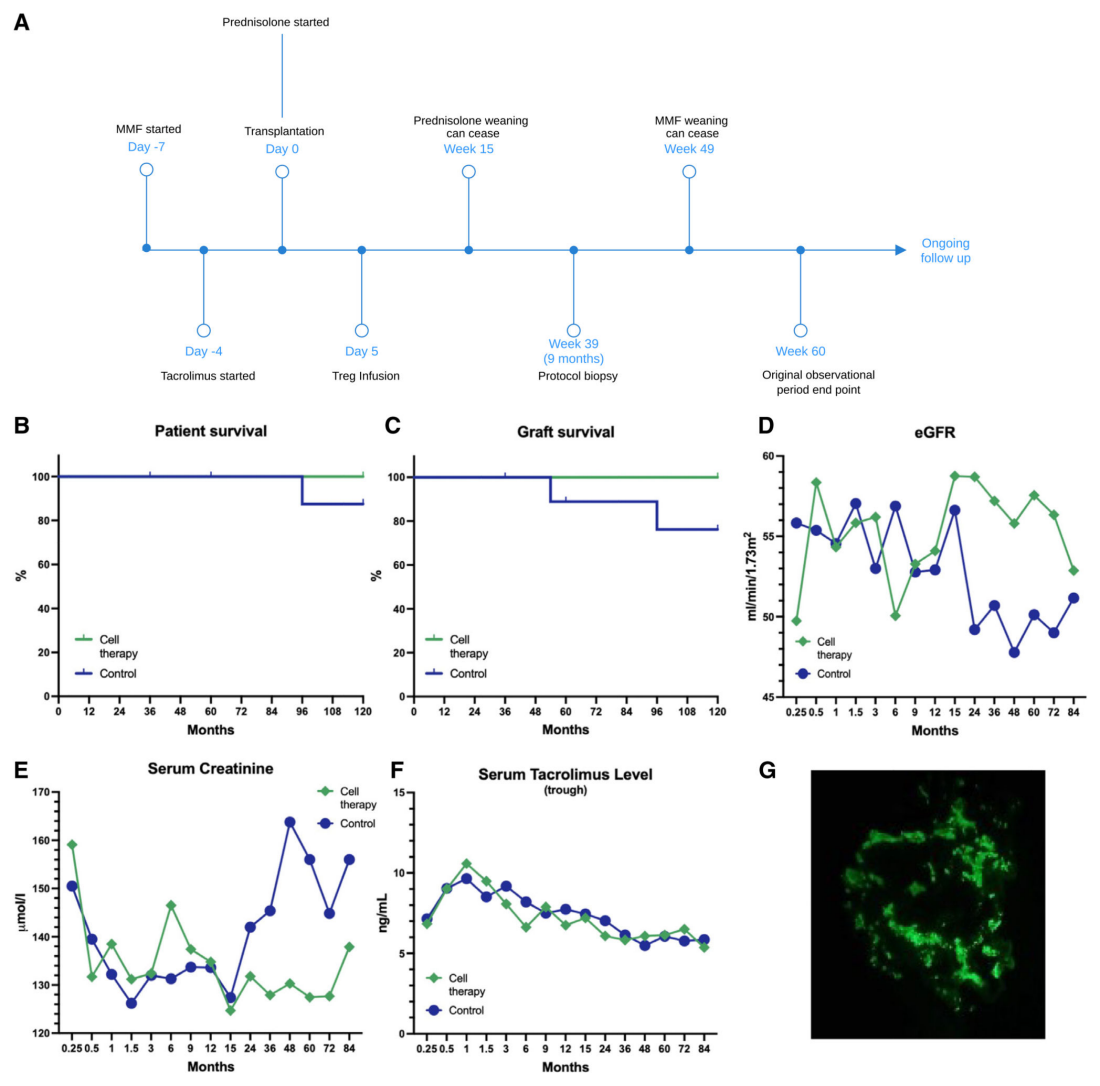
Study timeline and clinical outcome data (A) ONE Study overview schematic outlining commencement and initiation of weaning of immunosuppressive therapy in the Treg therapy group. Treg adoptive transfer was performed in the cell therapy group at postoperative day 5. The individual cell doses for each patient are listed in Table S1. (B) Patient survival in the reference (*n* = 10) and cell therapy groups (*n* = 10) up to 120 months post-transplant. Censoring was performed if patients moved care providers. (C) Graft survival in the reference and cell therapy groups up to 120 months post-transplant. (D) Mean estimated glomerular filtration rate (eGFR) in reference patients and cell therapy patients up to 84 months post-transplant. eGFR was calculated using the 2009 CKD-EPI equation.^20^ (E) Mean serum creatinine level in reference and cell therapy patients up to 84 months post-transplant. (F) Mean tacrolimus level in reference and cell therapy patients up to 84 months post-transplant. Target tacrolimus levels were as follows: 3–12 ng/mL in the first 2 weeks following transplantation, 3–10 ng/mL between postoperative weeks 2 and 12, 3–8 ng/mL between postoperative weeks 12 and 36, and 3–6 ng/mL after postoperative week 36. (G) Representative immunofluorescence of a glomerulus demonstrating granular to globular deposits of IgA in the mesangium, illustrating recurrence of IgA nephropathy.

In the multicenter ONE Study, each center implemented a different type of cellular therapy for their patient cohort alongside a reference group receiving standard of care immunosuppression.^18^ Patients in the Oxford Treg trial have now completed 7 years of follow-up (Figures 1A and S1A; Table S1; *n* = 10 cell therapy and *n* = 10 reference group). At this 7-year mark, the Treg arm reports 100% patient and graft survival compared with 90% patient survival and 80% graft survival in the standard-of-care arm (Figures 1B and 1C). While renal function was comparable between the groups within the first 12 months, a decline in renal function was observed within the standard-of-care arm between 1 and 2 years after transplantation, which was not seen in the Treg therapy arm (Figures 1D, 1E, S1B, and S1C). Among the patients who discontinued mycophenolate mofetil (MMF) and transitioned to tacrolimus monotherapy, this regimen has been sustained for more than 7 years without any rejection episodes. Serum trough levels of tacrolimus showed no difference between patients receiving Tregs and those on standard immunosuppression throughout the follow-up period, with no increased tacrolimus burden in those on monotherapy (Figures 1F and S1D).

Over the 7-year period, no patients in either the cell therapy or reference groups developed *de novo* donor-specific antibodies (DSAs). Of the 10 patients treated with Tregs, eight underwent protocol biopsies at 9 months post-transplant. All biopsies showed focal lymphocytic infiltrates within the renal interstitium, yet this occurred against a backdrop of stable clinical presentations. Despite these infiltrates, three patients were successfully transitioned to tacrolimus monotherapy. Throughout the follow-up period, nine of the ten patients did not require additional biopsies. One exception was a patient whose baseline creatinine levels increased from 140 to 180 μmol/L. A subsequent biopsy suggested a possible recurrence of immunoglobulin (Ig)A nephropathy (Figure 1G), the primary underlying pathology, but without evidence of acute rejection or lymphocytic infiltrates and minimal chronic damage.

### Infused Tregs are transcriptionally distinct from circulating Tregs

To assess whether tissue findings correlated with long-term changes in the peripheral Treg compartment, we conducted simultaneous transcriptional and cell surface protein profiling of the Treg therapy product (termed TR001) and circulating Tregs (defined as CD127^low^CD25^hi^ CD4^+^ T cells) isolated from peripheral blood (PB) of four patients receiving Treg therapy.

**Figure 2:**
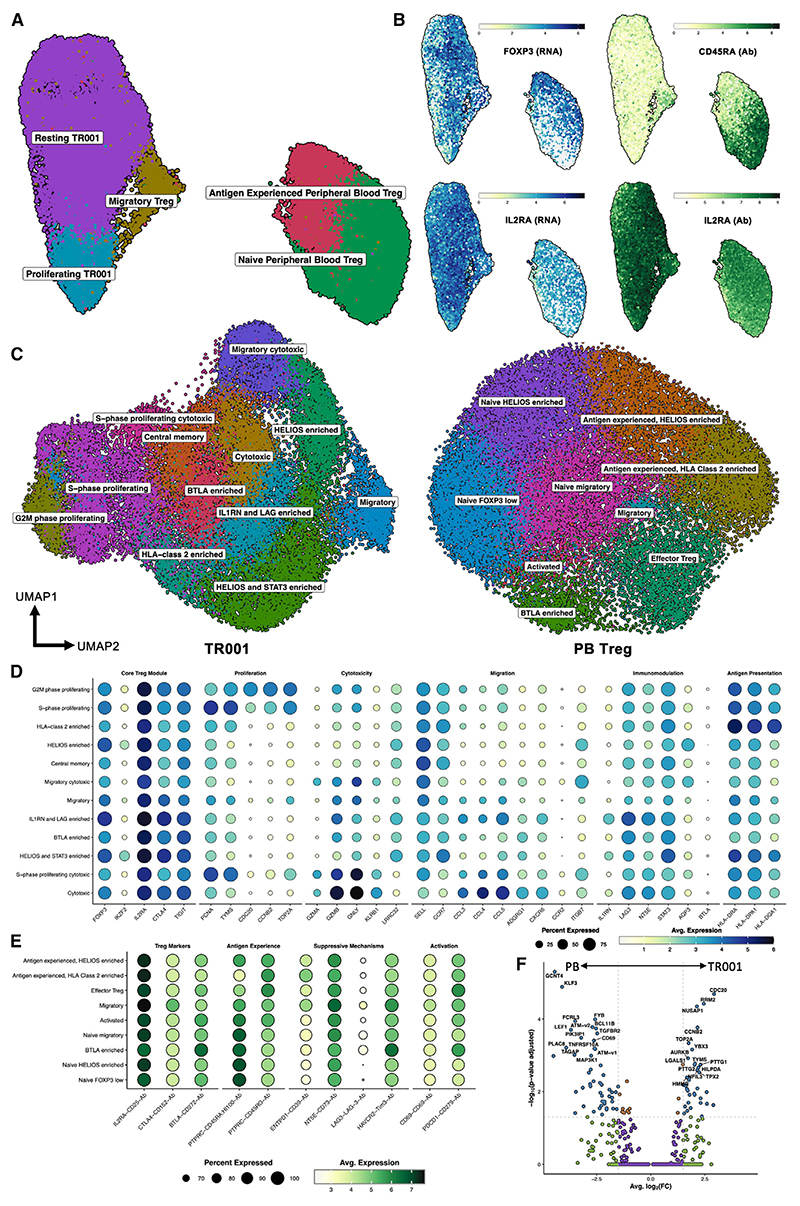
Phenotyping of adoptively transferred and peripherally circulating Tregs Probe-based single-cell RNA sequencing of adoptively transferred Tregs (TR001, *n* = 4) and paired peripherally circulating Tregs from 24 and 60 weeks post-transplant (*n* = 4) was performed with simultaneous quantification of 54 cell surface proteins. (A) Unsupervised clustering of adoptively transferred and peripherally circulating Tregs annotated by broad cluster type. (B) Expression of key Treg phenotypic markers FOXP3 and CD25 (protein and RNA) and the CD45 splice variant CD45RO. (C) Unsupervised clustering and annotation of adoptively transferred Tregs (TR001, left) and peripherally circulating Tregs (right). (D) Average expression and percentage expression of key Treg transcript modules across each TR001 cluster. (E) Average expression and percentage expression of key Treg protein marker modules across each PB Treg cluster. (F) Pseudobulk analysis (per patient) of TR001 and PB Tregs.

With the exception of one specific cluster, Tregs from the cell therapy product (TR001) and Tregs from PB (PB Tregs) clustered separately, indicating distinct transcriptional profiles between the sample sources (Figure 2A). The shared cluster, containing both TR001 and PB Tregs, was identified as migratory Tregs due to their protein expression of several chemokine receptors (Figures S2A and S2B). TR001 Tregs showed higher expression of *FOXP3* and *IL2RA* than PB Tregs (Figure 2B, left), which was supported by higher cell surface expression of CD25 (Figure 2B, bottom right). Expression of *FOXP3* was consistently high across all TR001 clusters, and an average of 20% co-expressed HELIOS (Figures S1E and S1F). PB Tregs clustered broadly into antigen naive and antigen experienced Tregs, as defined by their expression of CD45RA and CD45RO (Figures 2B and S1G), with significant proportions co-expressing FOXP3 and HELIOS (67% and 72% in naive and memory Tregs, respectively; Figures S1H and S1I). TR001 Tregs uniformly expressed several key Treg phenotypic and functional markers, including CTLA-4, CD39, CD73, and TIM3 (Figure S2C), and could further be characterized by their state of proliferation (Figure S2D).

To generate a detailed characterization of within-sample heterogeneity, TR001 and PB Tregs were integrated, dimensionally reduced, and clustered separately (Figures 2C and S2E). All TR001 Tregs consistently exhibited a core Treg gene signature (Figure 2D). Further re-clustering revealed additional diversity, enabling refined subclassification. Within the proliferating cluster, subclusters representing cells in the G2/M and S phases of the cell cycle were distinguishable using a composite cell cycle scoring approach (Figures 2D and S2D).

In the population initially annotated as resting TR001, further clusters were delineated based on the expression of *IKZF2* (encoding HELIOS), genes associated with migratory function, or transcripts indicative of Treg functionality (Figures 2C and 2D). Two of these clusters were notable for enriched *IKZF2* expression, with one also displaying higher levels of the transcription factor *STAT3*. Cytotoxic effector Tregs were identified across three clusters, characterized by their enrichment in granzyme B and granulysin, with markers for proliferation and migration such as S-phase proteins (*PCNA* and *TYMS*) and integrins, respectively. One cluster was distinguished by an abundance of human leukocyte antigen (HLA) class II transcripts, suggesting activation or enhanced antigen presentation capacity, and another by increased expression of LAG3 and the IL-1 decoy receptor IL-1RN. Central memory Tregs were marked by the co-expression of transcripts for CD62L and CCR7 (*SELL* and *CCR7*). The proportions of each cluster were comparable between patients except for one patient who exhibited a smaller HELIOS-enriched compartment and a separate patient who exhibited an enlarged migratory cytotoxic compartment (Figure S2G). This diversity within manufactured Tregs, despite a consistent core Treg signature, highlights the complexity of the infused product.

In the re-clustered PB Tregs (Figure 2C), nine subclusters were identified, split between four antigen-naive and five antigen-experienced groups (Figure 2E). All showed uniformly high surface expression of CD25 (Figure 2E). Within the naive clusters, one was marked by enriched cell surface expression of BTLA and TIM3 and a second by enriched HELIOS expression (Figures 2E and S2F). Among the antigen-experienced clusters, two showed enrichment for HELIOS, one for HLA class II, and one for expression of cytotoxic transcripts and KLRB1. Pseudobulk analysis comparing TR001 with circulating Tregs revealed increased proliferation-associated transcripts in TR001, while PB Tregs showed higher expression of the activation markers CD69 and TAGAP (Figure 2F). The relative proportions of each PB Treg subcluster were largely consistent across patients (Figure S2H). Notably, expression of *IL2*, a hallmark of effector T cell function, was absent in both PB and TR001 Tregs (Figure S2I).

### Focal lymphocytic infiltrates within transplant biopsies following Treg therapy are distinct from those in acute T cell-mediated rejection

**Figure 3:**
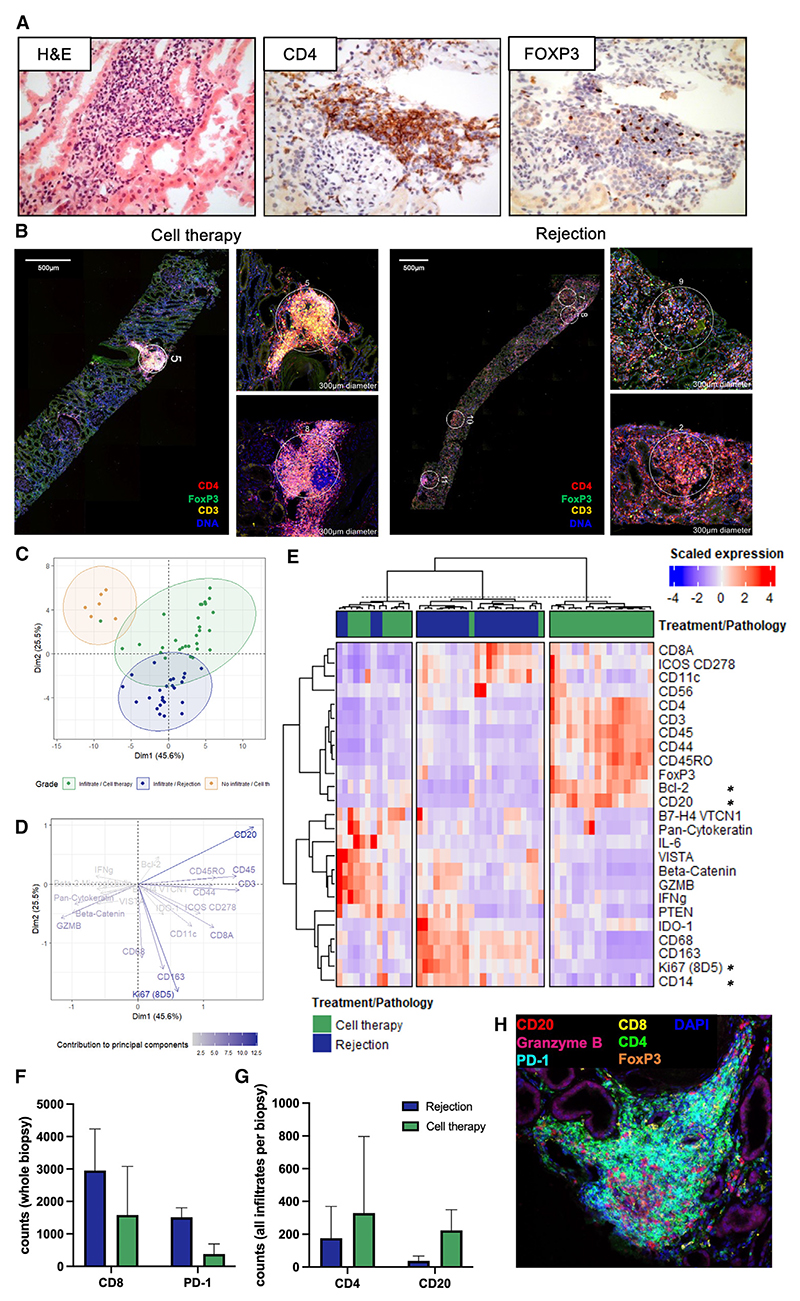
Spatial proteomics of immune infiltrates following Treg adoptive transfer Probe-based evaluation of 41 protein targets using the NanoString GeoMx Digital Spatial Profiling platform on formalin-fixed, paraffin-embedded (FFPE) renal core biopsies from both Treg-therapy-treated patients (*n* = 3) and patients experiencing acute T cell-mediated rejection (*n* = 2). (A) Representative hematoxylin and eosin staining (left), CD4 immunohistochemical staining with DAB (middle), and FOXP3 immunohistochemical staining with DAB (right). (B) Immunofluorescent staining of a representative immune infiltrate in a Treg-therapy-treated patient kidney transplant biopsy (left) versus the diffuse immune infiltrate seen in the transplant biopsy of a patient experiencing acute T cell-mediated rejection (right). Slides were stained with a DNA stain (SYTO 13), CD3 (Cy3), FOXP3 (Cy5), and CD4 (Cy7). Inset images illustrate example region-of-interest selections. (C) Dot plot illustrating the first and second dimensions of variance following principal-component analysis (PCA) representing the main difference between sample types. (D) PCA loading plot illustrating the contribution of the top 20 protein markers to the variance explained by the first and second dimensions. (E) Hierarchically clustered heatmap of the scaled expression of the top 25 markers with the highest variance across regions with significant cellular infiltrate (* indicates which markers have non-adjusted *p* values <0.05 when rejection and cell therapy infiltrates are compared). (F) Enumeration of CD8^+^ cells and PD-1^+^ cells across transplant biopsies from patients treated with Treg therapy (*n* = 3) or experiencing acute T cell-mediated rejection (*n* = 2) using multiplexed immunofluorescence. (G) Enumeration of CD4^+^ cells and CD20^+^ cells within observed lymphocytic infiltrates in transplant biopsies from patients treated with Treg therapy (*n* = 3) or experiencing acute T cell-mediated rejection (*n* = 2). Error bars across (F) and (G) represent mean ± standard deviation. (H) Representative 7-color immunofluorescence of a cell-therapy-associated cell infiltrate (red: CD20, pink: granzyme B, aqua: PD-1, yellow: CD8, green: CD4, orange: FOXP3, blue: DAPI).

We next sought to interrogate the composition and functionality of immune infiltrates within the transplanted kidneys of patients receiving TR001. Biopsies taken 9 months following Treg therapy from eight patients revealed focal lymphocytic infiltrates. These focal lymphocytic infiltrates were noted in the context of stable renal function and were distinct from those seen in acute cellular rejection. The infiltrates were predominantly identifiable within interstitial areas, often associated with fibrotic regions, and contained FOXP3^+^ cells indicative of Treg presence (Figure 3A).

To further analyze these infiltrates, we utilized a spatial profiling method that allowed for the simultaneous detection of 41 protein targets within specific regions of the biopsy. We compared biopsies showing cell-therapy-associated infiltrates (35 regions: 29 with infiltrates and six without from three biopsies) to those from patients experiencing acute T cell-mediated rejection (24 regions from two biopsies). Immunofluorescence highlighted the distinct morphology of the infiltrates associated with cell therapy—focal as opposed to the diffuse infiltrates, typical of rejection episodes (Figure 3B). Areas dense with immune cells, identified by high CD3, CD4, or FOXP3, were specifically selected for further analysis (Figure 3B, encircled regions).

After normalization (Figure S3A) and filtering against isotype negative controls, principal-component analysis underscored the differences in density and composition between infiltrates in Treg therapy compared with reference patients (Figures 3C and S3B). Cell therapy infiltrates were characterized by an enhanced expression of Treg-associated proteins (FOXP3, CD3, CD4, CD45RO), the B lymphocyte antigen CD20, the adhesion molecule CD44, and the apoptosis regulator Bcl-2 (Figures 3D and 3E). In contrast, infiltrates in T cell-mediated rejection were characterized by proliferative cytotoxic T lymphocyte (CD8A, Ki67) and inflammatory myeloid signatures (CD11c, CD14, CD68, CD163), as highlighted by hierarchical clustering (Figures 3E and S3C). Importantly, these observations were also demonstrable on multiplexed immunofluorescence staining, where a higher number of CD8^+^ T cells characterized biopsies exhibiting acute T cell-mediated rejection (Figure 3F), while immune infiltrates were enriched for CD4^+^ T cells and CD20^+^ B cells (Figures 3G and 3H).

These findings suggest that infiltrates associated with Treg therapy are not only phenotypically but also compositionally distinct from those observed in rejection. They show enhanced involvement of B cells and pro-survival signaling within the lymphocytic population, potentially indicating an immunomodulatory effect mediated by the infused Tregs.

### Spatial transcriptomics confirms the presence of infiltrating Tregs and identifies a regulatory gene expression signature that characterizes infiltrates

To investigate the gene expression programs characterizing immune infiltrates after Treg therapy, we utilized a spatial transcriptomics approach. This method allowed us to locate and quantify 84 specific genes of interest, comparing cell-therapy-associated infiltrates (58 regions from 7 biopsies) with protocol transplant biopsies (61 regions from 5 biopsies) from non-rejecting control patients and biopsies from patients experiencing acute T cell-mediated rejection (52 regions from 4 biopsies). Protocol and acute T cell-mediated rejection biopsies were not available from within the ONE Study reference group, and therefore archival age and immunosuppression-matched kidney transplant biopsies were utilized. After data normalization (Figure S3D) and filtering of negative probes, we found that the density of cellular infiltrates was similar across all groups (Figure S3E). Principal-component analysis clearly separated biopsy type (Treg therapy, T cell-mediated rejection, or protocol biopsies) and degree of immune infiltrate (Figures S3F and S3G).

**Figure 4:**
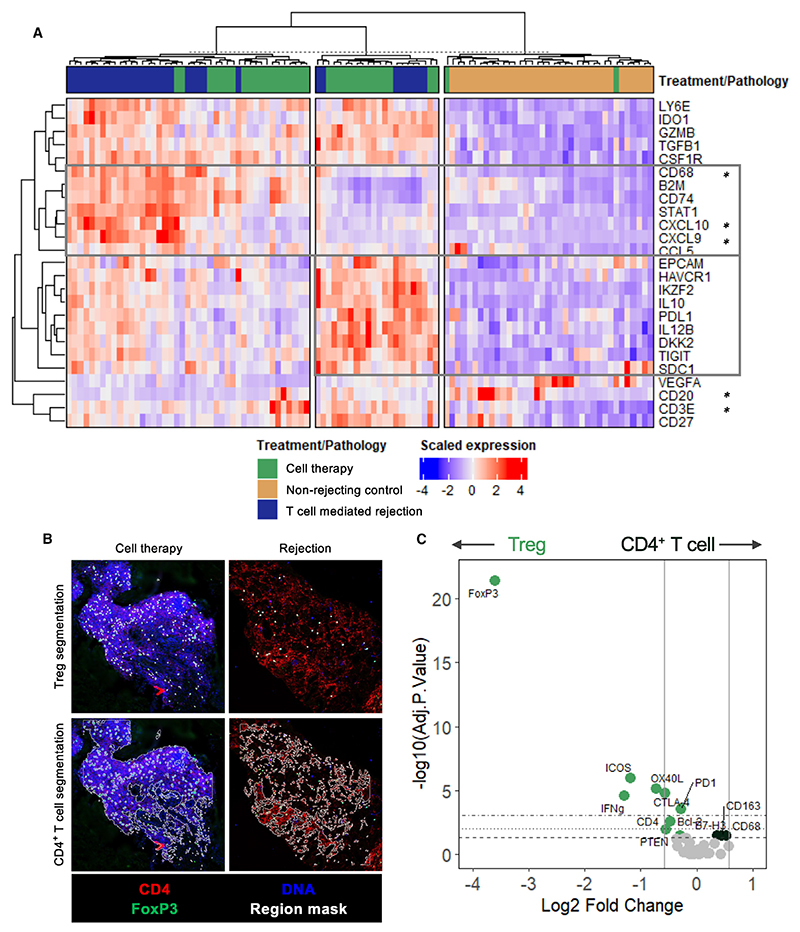
Spatial transcriptomics of immune infiltrates following Treg adoptive transfer Probe-based evaluation of 84 RNA targets of interest using the NanoString GeoMx Digital Spatial Profiling platform on FFPE renal core biopsies from either Treg-therapy-treated patients (“Cell Therapy,” *n* = 7), patients experiencing acute T cell-mediated rejection (“T cell-mediated rejection,” *n* = 4), or protocol biopsies from renal transplant recipients under standard-of-care immunosuppression (“Non-rejecting control,” *n* = 5). (A) Hierarchically clustered heatmap of the scaled expression of the top 25 markers with the highest variance across regions of interest containing cellular infiltrates. One region of interest was excluded due to anomalous high expression despite normalization. Boxes denote the myeloid rejection signature (top box: CD68, CXCL9, CXCL10) and enriched expression of immunoregulatory and checkpoint molecules within cell therapy biopsies (bottom box: IKZF2, IL-10, PDL1, TIGIT). (B) Illustrative rare cell masking strategy. Tissues were stained for immunofluorescence with a DNA stain (SYTO 13), FOXP3 (Cy5), and CD4 (Cy7). Immune-dense regions were selected on the density of DNA-stained nuclei containing positive staining of CD4 and FOXP3. Tregs were identified by co-expression of CD4 and FOXP3 (top). Surrounding immune-dense regions were identified by subtracting FOXP3^+^ cells from surrounding CD4^+^ regions (bottom). Representative immunofluorescence images of rare-cell-masked regions included in the analysis are provided in Figure S4I. (C) Differential expression of protein targets between Tregs and surrounding CD4^+^ cells. Markers highlighted with asterisks have adjusted *p* values <0.05.

Within cell-therapy-associated infiltrates, we identified a regulatory gene cluster including the canonical Treg marker HELIOS as well as known markers of Treg suppressive function (IL-10, PDL1, TIGIT). This was distinct from the pro-inflammatory gene signature that was found to be associated with rejection (CD68, B2M, CD74, CXCL9, CXCL10) (Figures 4A, S5H, and S5I). Additionally, CD20 was upregulated at a transcriptional level in cell therapy biopsies (Figures 4A and S5I).

To further assess the Treg phenotype within the transplant biopsies, we performed *in situ* spatial rare cell masking, whereby Tregs identified by co-expression of CD4 and FOXP3 are compared to surrounding CD4^+^ FOXP3^-^ regions (Figures 4B and S4A–S4G). Consistent with the immunofluorescence findings, FOXP3 protein was significantly upregulated, confirming Treg identity. Importantly, key Treg functional markers, including CTLA4, PD1, OX40L, and ICOS, were expressed more abundantly in these Tregs compared to surrounding CD4^+^ cells, indicating that Tregs maintain their functional phenotype within cell therapy aggregates (Figures 4C and S4H).

## Discussion

This extended follow-up of patients from the ONE Study, who received autologous Treg therapy, demonstrates not only excellent long-term transplant outcomes but also the distinctive nature of the immune infiltrates that are associated with Treg treatment. All patients maintained functional primary transplants up to 7 years post-transplant without induction immunosuppression, with three currently maintained on tacrolimus monotherapy. These findings highlight the potential of Treg therapy to achieve long-term transplant stability while reducing reliance on conventional immunosuppression.

Our analysis using single-cell RNA sequencing with paired antibody detection (CITE-seq) confirms that the good manufacturing practice (GMP)-compliant manufacturing process produces a Treg product with uniform expression of key regulatory genes and proteins, including CD25, CTLA-4, CD73, CD39, TIGIT, and TIM3.^21^ Interestingly, unsupervised clustering revealed subtle differences delineating Treg subsets, including effector Tregs, characterized by increased expression of cytotoxicity markers (granzyme A, granzyme B, and granulysin), BTLA-enriched Tregs, and Tregs enriched for transcripts encoding a decoy receptor for the pro-inflammatory cytokine IL-1 (IL1RN). Importantly, no production of IL-2 was observed, and similar proportions of proliferating and resting cells were present within each product. A population of FOXP3^low^ naive Tregs (CD45RA^+^) was detectable in Tregs isolated from the PB of patients, which nevertheless expressed core Treg-associated transcripts (IL-2RA, CTLA-4, TIGIT, and LAG-3). However, FOXP3 protein expression assessed by flow cytometry demonstrated markedly less pronounced differences in expression between naive and antigen-experienced Tregs. This may be related to differences in *FOXP3* translation between cell populations, technical artifacts, or a small population of contaminating effector cells. The latter is less likely, given the high expression of FOXP3 on flow cytometry alongside the expression of transcripts for other core Treg and immunomodulatory molecules.

Significantly, we have characterized the lymphoid infiltrates within transplanted kidneys following Treg therapy, finding them distinct from those observed during acute T cell-mediated rejection. These infiltrates, unlike those in rejection, do not compromise the graft but are associated with stable renal function and the ability to safely reduce systemic immunosuppression. Employing spatial profiling techniques, we find that these infiltrates are Treg enriched and also associated with a B cell signature. Infiltrates express an immunoregulatory gene signature including HELIOS, IL-10, PDL1, and TIGIT, while Tregs within these aggregates maintain expression of essential Treg markers such as FOXP3, ICOS, and CTLA-4. Interestingly, within infiltrates, we observed enrichment of interferon (IFN)γ in Tregs compared with surrounding CD4^+^ lymphocytes. Expression of IFNγ has been previously described in Tregs, where it has been shown to contribute to their induction and function.^22–24^ This contrasts sharply with the dense, disorganized infiltrates typical of acute rejection, which we found to be marked by cytotoxic, myeloid, and inflammatory signatures, which suggests the requirement for increased immunosuppression. The distinct morphology and benign nature of the infiltrates after Treg therapy represent a shift in how post-transplant infiltrates may be viewed. These data support a model in which Tregs may foster a local immunoregulatory environment conducive to immunosuppression minimization.

The presence of similar Treg-rich organized lymphoid structures (TOLSs) in the renal parenchyma of tolerized transplanted kidneys in mice,^25^ and their resemblance to the human transplant infiltrates we observe, provides a compelling comparative framework. Compositionally, TOLSs are rich in Tregs, B cells, and natural killer (NK) cells. A time-course single-cell RNA-seq analysis of tolerant mouse kidneys reveals that early post-transplant T cell dominance gradually shifts as the B cell population consolidates over time. This is driven by increasing proportions of follicular memory and transitional memory B cells observed alongside increased expression of IgA within the graft.^26,27^ Importantly, Treg depletion in this model results in prompt graft rejection. However, later deletion of Treg does not, suggesting that the dominant mechanism of tolerance may develop over time.

The findings observed in Treg-therapy-treated patients echo findings in other studies of transplant tolerance, where B cell signatures indicate a potentially tolerogenic role. In studies of operationally tolerant kidney transplant recipients, an increase in circulating transitional T1 and T2 B cell subsets that produced higher levels of IL-10 was identified.^28–30^ Concordantly, the risk of developing acute T cell-mediated rejection has more recently been associated with the IL-10:TNFα ratio in circulating T1 transitional B cells.^31^ An increase in marginal zone B cells in the PB following Treg therapy also hints at potential interactions between Treg and B cell subsets.^19^ In models of adoptive transfer of regulatory B cells (Bregs), an increase in Treg infiltration into the allograft is observed, suggesting a possible bidirectional effect.^32^ However, B cells are also identifiable in rejecting kidney transplants with polarization toward an inflammatory innate-like phenotype and production of antibodies against renal antigens.^33,34^ A similar innate B cell population has also been implicated in the pathogenesis of chronic rejection following lung transplantation.^35^ This dual nature of B cells in transplant dynamics highlights the complexity of the immune milieu following transplantation.

In summary, we present the first report of lymphocytic aggregates within the kidney transplants of patients receiving adoptive Treg therapy and provide a comparative characterization to biopsies from acute rejection and stable function under standard immunosuppression. The presence of these cellular infiltrates supports the idea that local immunoregulation may be a crucial component of the therapy’s effectiveness and capacity to facilitate immunosuppression minimization. Our ongoing phase 2b trial^36^ will further dissect these interactions, potentially paving the way for more targeted and effective cellular therapeutics.

## Limitations of the study

Our study is limited by the small number of patients in the initial phase 1 trial and sample availability, which precluded comparison to baseline circulating Tregs. Efforts to characterize the infiltrate morphology and elucidate their prognostic significance in a larger phase 2b study (ISRCTN: 11038572) are ongoing.^36^ Additionally, while experimental approaches to track Tregs *in vivo* are under development,^37^ our TR001 product is not genetically modified, preventing the direct tracking of Treg migration in patients after adoptive transfer with a unique marker.

## Resource Availability

### Lead contact

Further information should be directed to and will be fulfilled by the lead contact, Fadi Issa (fadi.issa@nds.ox.ac.uk).

### Materials availability

This study did not generate new unique reagents.

## Star★Methods

### Key Resources Table

**Table:** 

REAGENT or RESOURCE	SOURCE	IDENTIFIER
Antibodies		
Anti-CD3:BV510 (clone UCHT1)	BD Biosciences	Cat#563109; RRID: AB_2732053
Anti-CD25:PE (clone M-A251)	BD Biosciences	Cat#560989; RRID: AB_10563905
Anti-CD8:AF700 (clone HIT8a)	BioLegend	Cat#300920; RRID: AB_528885
Anti-CD127:PE-Cy7 (clone eBioDR5)	eBioscience	Cat#25-1278-42; RRID: AB_1659672
Anti-CD4:FITC (clone OKT4)	BioLegend	Cat#317408; RRID: AB_571951
Anti-CD27:BV605 (clone L128)	BD Biosciences	Cat#562656; RRID: AB_2744351
Anti-CD8:APC-Cy7 (clone RPA-T8)	BioLegend	Cat#301016; RRID: AB_314134
Anti-CD25: APC (clone M-A251)	BD Biosciences	Cat#560987; RRID: AB_10562034
Anti-CD25: APC (clone 2A3)	BD Biosciences	Cat#340907; RRID: AB_2819021
Anti-CD45RA: BV785 (clone HI100)	BioLegend	Cat#304140; RRID: AB_2563816
Anti-HLA-DR: AF700 (clone L243)	BioLegend	Cat#307626; RRID: AB_493771
Anti-TIGIT: PerCP eFluor710 (clone MBSA43)	eBioscience	Cat#46-9500-42; RRID: AB_10853679
Anti-CD70: FITC (clone 113-16)	BioLegend	Cat#355106; RRID: AB_2562280
Anti-CD4: BUV737 (clone SK3)	BD Biosciences	Cat#612748; RRID: AB_2870079
Anti-PD-1: PE (clone EH12.1)	BD Biosciences	Cat#560795; RRID: AB_2033989
Anti-IFNg: BV711 (clone 4S.B3)	BioLegend	Cat#502540; RRID: AB_2563506
Anti-HELIOS: BV421 (clone 22F6)	BioLegend	Cat#137234; RRID: AB_2565799
Anti-FOXP3: PE-eFluor 610 (clone PCH101)	Invitrogen	Cat# 61-4776-42; RRID: AB_2574610
Anti-FOXP3: PE-Dazzle 594 (clone 206D)	Biolegend	Cat# 320126; RRID: AB_2564025
Anti-Ki67: BUV395 (clone B59)	BD Biosciences	Cat#564071; RRID: AB_2738577
See Table S2 for AbSeq antibodies and clones	BD Biosciences	N/A
Biological samples		
PBMC aliquots (24- and 60-week samples)	ONE Study	NCT02129881
Renal transplant biopsies exhibiting regulatory T cell therapy associated infiltrates	ONE Study	NCT02129881
Renal transplant biopsies exhibiting acute T cell mediated rejection	Oxford Center for Histopathology Research (OCHRe Biobank)	N/A
Protocol renal transplant biopsies	Monash University	N/A
Chemicals, peptides, and recombinant proteins		
PharmLyse	BD Biosciences	Cat#555899
Fc block	BD Biosciences	Cat#564219
Critical commercial assays		
Human T cell Expression primer panel	BD Biosciences	Cat#633751
High Sensitivity dsDNA Kit	Thermo Fisher	Cat#Q33120
High Sensitivity D1000 ScreenTape	Agilent	Cat#5067-5584
Deposited data		
Single-cell RNA sequencing data (CITEseq)	This study	GSE281542
GeoMx spatial data (RNA/Protein)	This study	https://doi.org/10.5281/zenodo.14008377
Oligonucleotides		
See Table S2 for custom designed primer panel	BD Bioscience	N/A
Software and algorithms		
R 4.2.1	R Project for Statistical Computing	r-project.org
R 4.3.1	R Project for Statistical Computing	r-project.org
BD Rhapsody Analysis pipeline (v 1.12.1)	BD Biosciences	https://www.bdbiosciences.com/en-us/products/software/rhapsodysequence-analysis-pipeline
Bowtie2	Brook et al.^36^	https://github.com/BenLangmead/bowtie2
Seurat (v5.0.1)	Jacob et al.^37^	https://github.com/satijalab/seurat/
Batchelor (v1.18.0)	Levy et al.^20^	https://bioconductor.org/packages/release/bioc/html/batchelor.html
SCpubr (V2.0.2)	Langmead and Salzberg^38^	https://github.com/enblacar/SCpubr
Factoextra (v1.0.7)	The Comprehensive R Archive Network	https://cran.r-project.org/web/packages/factoextra/index.html
ComplexHeatmap (v2.12.1)	Hao et al.^39^	https://bioconductor.org/packages/release/bioc/html/ComplexHeatmap.html
Ime4(v1.1.30)	Haghverdi, Lun, Morgan and Marioni^40^	https://cran.r-project.org/web/packages/lme4/index.html
ImerTest (v3.1.3)	Blanco-Carmona^41^	https://cran.r-project.org/web/packages/lmerTest/index.html

## Experimental Model And Study Participant Details

### Human clinical trial participants

Recipients of living donor kidney transplants were enrolled in the ONE Study (NCT02129881), a safety and feasibility trial of expanded autologous regulatory T cells. Demographic data on sex, gender, age, and race were collected at enrollment by patient self-reporting. Socioeconomic status data were not collected. The trial adopted a standard 3 + 3 dose escalation design at 1, 3, 6, and 10 x 10^6^ cells/ kg expanded from three hundred and eighty milliliter of whole blood as previously described.^19^ Treg therapy was administered on postoperative day 5 through a central venous cannula. Patients treated with Treg therapy received UK standard of care immunosuppression comprising triple maintenance therapy of the calcineurin inhibitor tacrolimus, the antiproliferative agent mycophenolate mofetil, and a reducing dose of prednisolone. Induction immunosuppression with basiliximab was held due to its potential to interact with adoptively transferred Treg. Patients receiving Treg therapy underwent a protocol biopsy at 9 months post-transplant and the option to withdraw mycophenolate was available at this point at the discretion of the clinical team where no evidence of rejection was present on the biopsy.

## Method Details

### Clinical monitoring

Clinical and immune monitoring was undertaken as per clinical protocol, with trial visits prior to transplantation (V01), 1 week (V02), 2 weeks (V03), 4 weeks, (V04), 8 weeks (V05), 12 weeks (V06), 24 weeks (V07), 36 weeks (V08), 48 weeks (V09) and 60 weeks (V10) following transplantation. Serum creatinine and trough tacrolimus levels were measured as part of clinical care by the local hematology and biochemistry departments. eGFR was calculated using the CKD-EPI equation.^20^ After V10, follow up was arranged as per routine standard of care by the local clinical team including routine viral screening, assessment of renal function, and tacrolimus dose titration. DSA analysis was performed by the local NHS Blood and Transplant laboratory, with the standard diagnostic threshold for a positive assay defined as a median fluorescence intensity greater than 1000. All clinically relevant HLA antigens (HLA-A, -B, -C, -DR, -DP, -DQ) were evaluated.

### PBMC isolation

Immunomonitoring of trial participants was undertaken per protocol within the trial for the duration of follow up (60 weeks). Each immunomonitoring visit included a peripheral blood draw from which mononuclear cells were isolated and cryopreserved for downstream analysis. Briefly, between 10 and 30mL of blood was collected from a peripheral vein into an EDTA Vacutainer which was then transported to a central laboratory and processed the same day. PBMCs were isolated by density centrifugation whereby whole blood was diluted 1:1 with PBS, overlaid on Ficoll-Paque (density 1.077 g/mL), and centrifuged at 1105g for 30 min at room temperature. Cells were washed twice in PBS and red blood cells were lysed with BD Pharmlyse for 5 min at room temperature. After a final PBS wash, cells were enumerated with a hemocytometer and light microscope before cryopreservation in 45% fetal calf serum, 45% RPMI and 10% DMSO and cooled at 1°C/minute to −80°C before being transferred to the vapor phase of liquid nitrogen (−196°C) for long-term storage.

### Single cell RNA sequencing: cell preparation and capture

Cryopreserved aliquots of TR001 and paired PBMC samples isolated at 24-week and 60-week post-transplant from 4 patients selected randomly from patients with complete sample availability were thawed at 37°C and resuspended drop-by-drop in X-VIVO 15 (Lonza) with 1% heat-inactivated, filtered human AB serum (Sigma). Cells were then washed with cold PBS +2% FBS for cell counting on a hemocytometer. An aliquot of approximately 2 x 10^6^ cells was then taken for single-cell RNA-sequencing, and the remaining volume was taken for intracellular immunostaining.

Cells were washed with cold PBS +2% FBS and incubated for 20 min at 4°C with Fc block reagent (BD Biosciences) and a unique oligo-conjugated sample barcoding antibody (sample multiplexing kit; BD Biosciences). After this initial incubation, cells were stained with anti-CD3-BV510 (clone UCHT1, BD Biosciences), anti-CD25-PE (clone M-A251, BD Biosciences), anti-CD8-AF700 (clone HIT8a, BioLegend), anti-CD127-PECy7 (clone eBioDR5, eBioscience), and anti-CD4-FITC (clone OKT4, BioLegend) for 30 min at 4°C. Cells were then sorted in a BD FACSAria Fusion sorter (BD Biosciences). For the TR001 expanded product, ~25,000 cells were sorted per donor based on FSC and SSC profile to enrich for live viable cells. For the PBMC samples, ~ 18,000 cells were sorted per sample from the CD127^low^CD25^hi^ gate to enrich for the regulatory T cell population.

Following cell sorting, cells from the same donor (expanded Treg, 24-week PB Tregs, and 60-week PB Tregs) were pooled together, washed in cold BD stain buffer (BD Bioscience) and incubated with a combination of 54 AbSeq antibodies (BD Biosciences, Table S2) according to the manufacturer’s instructions. Cells were then washed three times in cold BD sample buffer (BD Biosciences), filtered and enumerated with a hemocytometer and light microscope. Each cell pool was then resuspended in 620μL of cold sample buffer at a final concentration of 40 cells/μL - for an estimated capture rate of ~20,000 single-cells - and immediately loaded on one BD Rhapsody cartridges (BD Biosciences) for single-cell capture.

### Single cell RNA sequencing: cDNA library preparation and sequencing

Single-cell capture and cDNA library preparation was performed using the BD Rhapsody Express single-cell analysis system (BD Biosciences), according to the manufacturer’s instructions. cDNA was initially amplified for 11 cycles (PCR1) using the pre-designed Human T cell Expression primer panel (BD Biosciences) containing 259 primer pairs, together with a custom designed primer panel containing an additional 806 primer pairs (BD Biosciences). A total of 1,065 primer pairs targeting 971 different genes were targeted for amplification with this targeted panel (see key resources table for gene content). The purified mRNA and Sample Tag PCR1 products were further amplified (10 cycles). The concentration, size and integrity of the resulting PCR 2 products were assessed using both Qubit (High-Sensitivity dsDNA Kit; Thermo Fisher) and the Agilent 4200 TapeStation system (High Sensitivity D1000 ScreenTape; Agilent). The final products were adjusted to 2.5 ng/μL (mRNA), 1.1 ng/μL (Sample Tag) and 0.7 ng/μL (AbSeq) and underwent a final round of amplification (6 cycles for mRNA and Sample Tag and 7 cycles for AbSeq) using indexes for Illumina sequencing to prepare the final libraries. Final libraries were quantified using Qubit and Agilent TapeStation and pooled (~61/35/4% mRNA/AbSeq/Sample Tag ratio) to achieve a final concentration of 5 nM. Final pooled libraries were sequenced (150 bp paired-end) on a NovaSeq 6000 (Illumina).

### Intracellular immunostaining

Intracellular immunostaining was performed on the same samples used for single-cell RNA-sequencing. Following cell wash with cold PBS +2% FBS, cells were immunostained with fluorochrome-conjugated antibodies against surface-expressed markers for 45 min at 4°C: anti-CD27-BV605 (clone L128, BD Biosciences), anti-CD8-APC-Cy7 (clone RPA-T8, BioLegend), anti-CD25-APC (clone 2A3, BD Biosciences), anti-CD25-APC (clone M-A251, BD Biosciences), anti-CD45RA-BV785 (clone HI100, BioLegend), anti-HLA-DR-AF700 (clone L243, BioLegend), Anti-TIGIT-PerCP-eFluor710 (clone MBSA43, eBioscience), anti-CD70-FITC (clone 113-16, BioLegend), anti-CD4-BUV737 (clone SK3, BD Biosciences), anti-PD-1-PE (clone EH12.1, BD Biosciences). Following a further wash, fixation and permeabilization was performed using the FOXP3 Fix/Perm Buffer Set (eBioscience) according to the manufacturer’s instructions. Cells were then immunostained with fluorochrome-conjugated antibodies against intracellular markers for 45 min at room temperature: anti-IFNγ-BV711 (clone 4S.B3, BioLegend), anti-HELIOS-BV421 (clone 22F6, BioLegend), anti-FOXP3-PE-eFluor-610 (clone PCH101, Invitrogen), anti-FOXP3-PE-Dazzle594 (clone 206D, BioLegend), anti-Ki67-BUV395 (clone B59, BD Biosciences). Immunostained samples were acquired using a BD Fortessa (BD Biosciences) flow cytometer with FACSDiva software (BD Biosciences) and analyzed using FlowJo (Tree Star, Inc.).

### Histology, immunohistochemistry, and immunofluorescence

Renal transplant biopsies were taken nine months following transplantation as per the clinical protocol. Briefly, following infiltration of local anesthetic, a renal transplant biopsy under ultrasound guidance was taken with a 16G core biopsy needle. Samples were immediately transferred to 10% neutral buffered formalin and transported to a central processing laboratory where they were dehydrated and embedded in paraffin. 5μm sections were acquired and haematoxylin and eosin staining or immunohistochemistry were performed using the LeicaBOND clinical-grade automated staining system. Slides were scanned and reported by the central trial pathologist. Biopsies with histological evidence of acute T cell mediated rejection, matched for immunosuppression exposure and time post-surgery were identified by manual screening of locally available clinical samples (Oxford Radcliffe Biobank). Routine (not for-cause) surveillance transplant biopsies were not available locally and therefore surveillance biopsies, matched for immunosuppression exposure and time post-surgery, were identified by manual screening of available clinical samples at a second center (Monash University). Multispectral immunofluorescence was performed on the Vectra Polaris platform staining for CD8 (Opal Polaris 480), CD4 (Opal 520), FOXP3 (Opal 570), CD20 (Opal 620), Granzyme B (Opal 690), and PD1 (Opal 780). Immunofluorescence of glomerular IgA deposition was performed by the clinical histopathology department at the John Radcliffe Hospital.

### Spatial proteomics and spatial transcriptomics

Assays were performed through the pre-commercial technology access program by NanoString. Briefly, 5μm sections were acquired onto Superfrost Plus slides and were baked at 60°C prior to deparaffinization in CitriSolv. Antigen retrieval was performed in citrate buffer (pH 6) for 15 min in a pressure cooker before blocking for 1 h in a humidity chamber. An antibody cocktail targeting 43 proteins of interest (AKT, B7-H3, B7-H4 VTCN1, Bcl-2, β2-microglobulin, pSTAT3, β-catenin, CD11c, CD14, CD163, CD20, STING TMEM173, CD3, CD4, CD44, CD45, CD45RO, VISTA, CD56, CD66B, CD68, CD8A, CTLA-4, CXCL9, FOXP3, GZMB, Histone H3, HLA-DR, ICOS CD278, IDO-1, IFNγ, IL6, Ki-67, Mouse IgG, OX40L CD252 TXGP1, *p*-ATK, PanCK, PD-1, PD-L1, PTEN, Rabbit IgG, S6, STAT 3) and 3 morphology markers (CD4, FOXP3, CD3) was incubated on the tissue overnight. Antibodies were subsequently fixed in 4% PFA for 30 min, nuclei were stained with SYTO13 for 15 min and were acquired on the GeoMx platform. Targets were identified and quantified using the nCounter system. Following collection on the GeoMx platform, oligo pools from individual ROIs were hybridized with reporter probes and pooled and then loaded onto the nCounter.

For quantification of RNA targets, 5μm sections were mounted on Superfrost Plus slides under RNAse free conditions. After baking at 60°C, tissues were deparaffinized in Citrisolv and rehydrated. Antigen retrieval was performed for 15 min in Tris-EDTA buffer (pH 9) before exposure of RNA targets by incubation at 37°C in proteinase K solution (1 μg/mL) for 15 min. Tissues were then fixed in 10% neutral buffered formalin before *in situ* hybridization overnight with a probe cocktail targeting 84 transcripts of interest (4-1BB, AKT1, ARG1, B2M, B7-H3, BATF3, BCL2, CCL5, CCND1, CD11b, CD11c, CD20, CD27, CD3E, CD4, CD40, CD40LG, CD44, CD45, CD47, CD68, CD74, CD86, CD8A, CMKLR1, CSF1R, CTLA4, CTNNB1, CXCL10, CXCL9, CXCR6, DKK2, EPCAM, FAS, FOXP3, GZMB, HAVCR1, HIF1A, HLA-DQA1, HLA-DRB, HLA-E, ICAM1, ICOSLG, IDO1, IFNAR1, IFNG, IFNGR1, IKZF2, IL10, IL12B, IL15, IL6, ITGAV, ITGB2, ITGB8, KI67, LAG3, LY6E, Multi KRT, NKG7, NRP1, OAZ1, pan-Melanocyte, PD1, PDL1, PDL2, PECAM1, POLR2A, PSMB10, PTEN, RAB7A, SDC1, SDHA, STAT1, STAT2, STAT3, TBX21, TGFB1, TIGIT, Tim3, TNF, UBB, VEGFA, VISTA). The tissues were stained with morphology markers against CD3 (Cy7), CD20 (Cy5) and DNA (SYTO13) for 1 h before acquisition on the GeoMx platform. Following collection on the GeoMx platform, oligo pools from individual ROIs were hybridized with reporter probes and pooled and then loaded onto the nCounter.

## Quantification And Statistical Analyses

### Analysis of single cell RNA sequencing data

Raw fastq files were processed initially using the BD Rhapsody Analysis pipeline. Briefly, low quality read pairs were filtered by length (R1<60bp, R2<42bp), quality score (R1 or R2 <20), and base complexity (single nucleotide frequency ≥0.55 for R1 or ≥0.8 for R2). R1 reads were annotated to identify cell IDs and UMIs and R2 reads were mapped to reference panel sequences using Bowtie2.^38^ Valid R1 and R2 pairs were then filtered using recursive substitution error correction (RSEC) to mitigate UMI errors whereby molecules differing by one base within the UMI sequence are amalgamated. Count matrices were loaded into Seurat (5.0.1) and initial filtering was performed by removing cells expressing fewer than 60 genes or with fewer than 100 transcripts, determined empirically by count distributions, or if cells were assigned as multiplets based on antibody hashing.^39^ RNA data were log normalized and scaled and variable features identified with default parameters. Antibody data were normalized using the centered log ratio normalization method. The number of principal components to take forwards was determined empirically via scree plot visualization. Data were integrated using FastMNN^40^ and neighbors and clusters were identified with a resolution of 1. Conserved markers were identified with the FindConservedMarkers with a log fold threshold of 0.25. Clusters were annotated by expression of canonical and phenotypic marker genes ranked by fold change between clusters. PB and TR001 data were split by hashing antibody expression and re-processed separately using the same pipeline. Proliferation scoring was calculated by a module score of the cell cycle genes PCNA, TYMS, MCM4, CDCA7, RRM2, HMGB2, CDK1, NUSAP1, UBE2C, BIRC5, TPX2, TOP2A, NDC80, MKI67, CENPF, TACC3, CCNB2, CKAP2, AURKB, CDC20, KIF2C, HMMR, and AURKA. Visualization was performed with Seurat and SCpubr.^39,41^

### Analysis of spatial proteomic and spatial transcriptomic data

To assure the technical quality of nCounter readouts, ROIs were excluded if their binding density was outside of 0.05–2.25 spots/μm^2^ or their positive ERCC control scaling factors outside of 0.3–3. Counts were normalized to the housekeeping protein (Histone H3 and S6) or housekeeping genes (POLRA2, RAB7A, SDHA) expression. Principal component analyses identified the major sources of variance (R 4.2.1, R *factoextra* version 1.0.7). Hierarchical clustering of Pearson coefficients was applied to scaled protein expressions (*ComplexHeatmap* version 2.12.1^42^). Differential protein/RNA expression of whole ROIs was assessed by applying linear mixed models accounting for tissue origin as a random variable onto log transformed data (*lme4* version 1.1–30^43^; *lmerTest* version 3.1–3^44^).

Details of statistical analyses including sample number (n), statistical test employed, and descriptive statistics are described in the corresponding figure legends and results text.

## Additional Resources

Clinical Trial Reference Number: NCT02129881.

## Supplementary Material

Supplemental information can be found online at https://doi.org/10.1016/j.medj.2024.11.014.

Supplemental Information

## Data Availability

Single-cell RNA-seq data have been deposited at GEO and are publicly available as of the date of publication under accession number GEO: GSE281542. GeoMx spatial transcriptomic data (RNA and protein) have been deposited in Zenodo (https://doi.org/10.5281/zenodo.14008377) and are publicly available as of the date of publication. Microscopy data reported in this paper will be shared by the lead contact upon request. Analyses in this study used pre-existing tools and did not generate original code. Any additional information required to reanalyze the data reported in this paper is available from the lead contact upon request.

## Highlights

- Patients receiving Treg therapy have excellent long-term outcomes
- Immune infiltrates within the transplant are observed after Treg therapy
- Infiltrates are enriched for regulatory genes including *IKZF2, IL10, PD-L1*, and *TIGIT*
- B cells are prominently enriched in infiltrates from Treg-treated patients

## In brief

McCallion et al. report that kidney transplant recipients treated with Treg therapy exhibit prominent immune infiltrates within the transplanted organ. Infiltrates occur in patients with excellent long-term outcomes and exhibit immunoregulatory and B cell signatures, supporting the notion that local regulation is a prominent mechanism of Treg therapy.

## Context and Significance

Kidney transplantation is the gold-standard treatment for end-stage renal disease, but it requires lifelong immunosuppression with substantial side effects. Treg therapy may help to reduce the reliance on conventional immunosuppression. In the current study, the authors show that renal transplant patients receiving Treg therapy had excellent long-term kidney function with no episodes of rejection over 7 years while also developing immune cell infiltrates within the graft. These infiltrates were enriched for immunoregulatory genes and B cells. These findings are significant for the interpretation of tissue responses to regulatory cell therapies and support the idea that local immunoregulation within the transplant may contribute to the effectiveness of Treg therapy.
