# Supplementary material for: Regulatory T cell therapy is associated with distinct immune regulatory lymphocytic infiltrates in kidney transplants
Source: Med. Author manuscript; Available in PMC 2025 Oct 10. (PMC7618234; doi:10.1016/j.medj.2024.11.014)
Supplement: Supplemental Information [file EMS209352-supplement-Supplemental_Information.zip › 1-s2.0-S2666634024004550-mmc1.pdf]

Med, Volume *6*

Supplemental information

Regulatory T cell therapy is associated with distinct immune regulatory lymphocytic infiltrates in kidney transplants

Oliver McCallion, Amy R. Cross, Matthew O. Brook, Conor Hennessy, Ricardo Ferreira, Dominik Trzupek, William R. Mulley, Sandeep Kumar, Maria Soares, Ian S. Roberts, Peter J. Friend, Giovanna Lombardi, Kathryn J. Wood, Paul N. Harden, Joanna Hester, and Fadi Issa

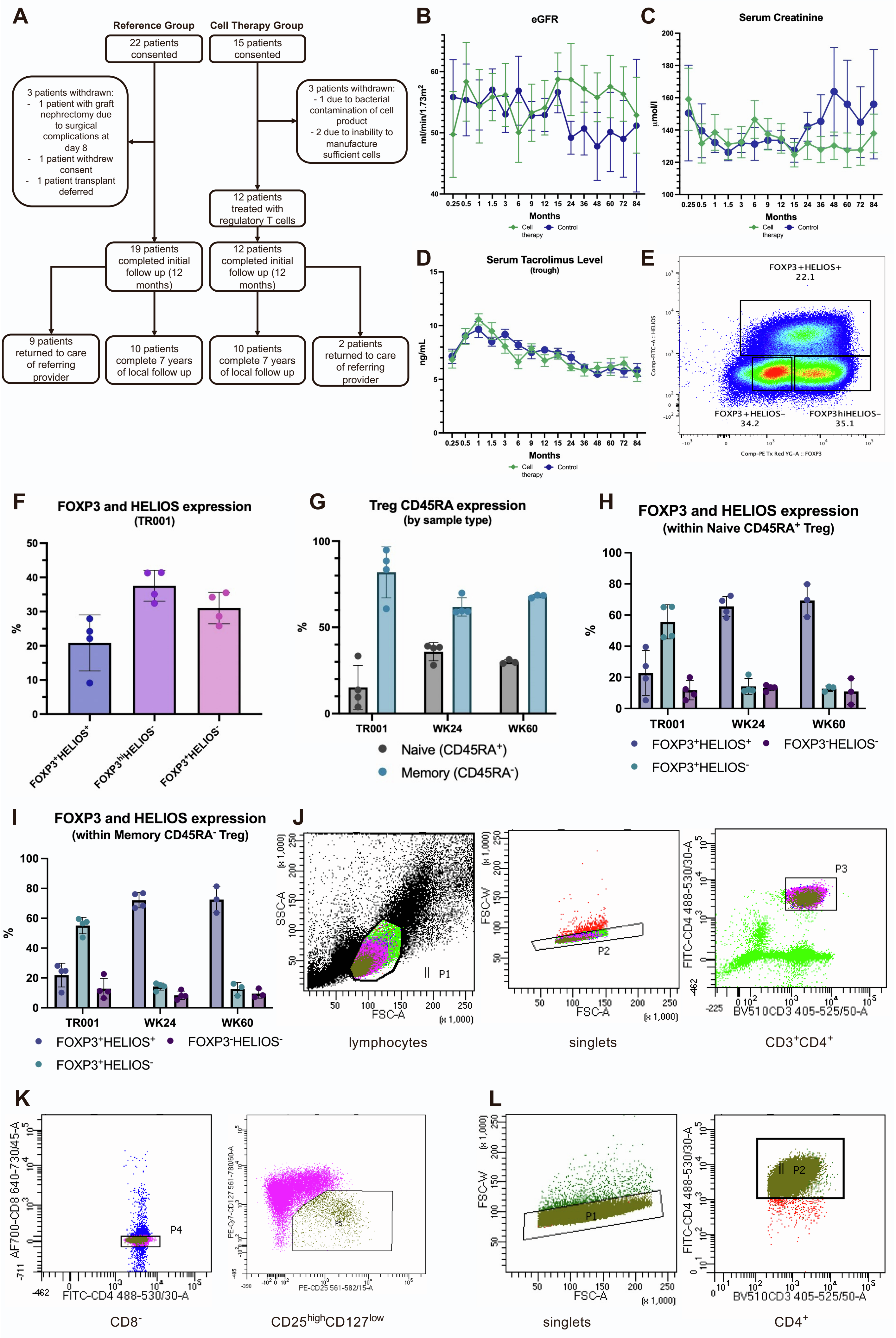
A
Reference Group
Cell Therapy Group
22 patients consented
15 patients consented
3 patients withdrawn:
- 1 patient with graft nephrectomy due to surgical complications at day 8
- 1 patient withdrew consent
- 1 patient transplant deferred
3 patients withdrawn:
- 1 due to bacterial contamination of cell product
- 2 due to inability to manufacture sufficient cells
12 patients treated with regulatory T cells
19 patients completed initial follow up (12 months)
12 patients completed initial follow up (12 months)
9 patients returned to care of referring provider
10 patients complete 7 years of local follow up
10 patients complete 7 years of local follow up
2 patients returned to care of referring provider
B
eGFR
ml/min/1.73m²
Months
Cell therapy
Control
C
Serum Creatinine
µmol/l
Months
Cell therapy
Control
D
Serum Tacrolimus Level
(trough)
ng/mL
Months
Cell therapy
Control
E
FOXP3+HELIOS+
22.1
FOXP3+HELIOS–
34.2
FOXP3hiHELIOS–
35.1
Comp-FITC-A :: HELIOS
Comp-PE Tx Red YG-A :: FOXP3
F
FOXP3 and HELIOS expression
(TR001)
%
FOXP3+HELIOS+
FOXP3hiHELIOS-
FOXP3+HELIOS-
G
Treg CD45RA expression
(by sample type)
%
TR001
WK24
WK60
Naive (CD45RA+)
Memory (CD45RA-)
H
FOXP3 and HELIOS expression
(within Naive CD45RA+ Treg)
%
TR001
WK24
WK60
FOXP3+HELIOS+
FOXP3-HELIOS-
FOXP3+HELIOS-
I
FOXP3 and HELIOS expression
(within Memory CD45RA- Treg)
%
TR001
WK24
WK60
FOXP3+HELIOS+
FOXP3-HELIOS-
FOXP3+HELIOS-
J
SSC-A
FSC-A
P1
lymphocytes
FSC-W
FSC-A
P2
singlets
FITC-CD4 488-530/30-A
BV510CD3 405-525/50-A
P3
CD3+CD4+
K
AF700-CD8 640-730/45-A
FITC-CD4 488-530/30-A
P4
CD8-
PE-Cy7-CD127 561-780/60-A
PE-CD25 561-582/15-A
CD25highCD127low
L
FSC-W
FSC-A
P1
singlets
FITC-CD4 488-530/30-A
BV510CD3 405-525/50-A
P2
CD4+

Supplementary Figure 1, related to Figure 1 and 2: Sorting strategy for isolation of Treg from PBMC aliquots and intracellular protein phenotyping.

(A) CONSORT diagram **(B)** Mean eGFR in reference patients and cell therapy patients up to 84 months post-transplant. eGFR was calculated using the 2009 CKD-EPI equation. **(C)** Mean serum creatinine level in reference and cell therapy patients up to 84 months post-transplant. **(D)** Mean tacrolimus level in reference and cell therapy patients up to 84 months post-transplant. Error bars across A-C represent mean ± standard error of the mean. **(E)** Representative gating strategy of TR001 aliquot displaying three gates: $FOXP3^{+}HELIOS^{+}$, $FOXP3^{hi}HELIOS^{-}$, and $FOXP3^{+}HELIOS^{-}$. **(F)** Quantification of FOXP3 and HELIOS expression across TR001. **(G)** Expression of CD45RA quantified by flow cytometry across each sample type. **(H)** Bar chart quantifying FOXP3 and HELIOS expression within naïve $CD45RA^{+}$ Treg. **(I)** Bar chart quantifying FOXP3 and HELIOS expression within memory $CD45RA^{-}$ Treg. **(J, K)** Representative sorting strategy for Treg from PBMC, gating (from left to right) on lymphocytes, singlets, $CD3^{+}CD4^{+}$ lymphocytes, $CD3^{+}CD4^{+}CD8^{-}$ lymphocytes, and finally $CD3^{+}CD4^{+}CD8^{-}CD25^{hi}CD127^{low}$ Treg. **(L)** Representative sorting strategy for TR001 ($CD4^{+}$ singlets).

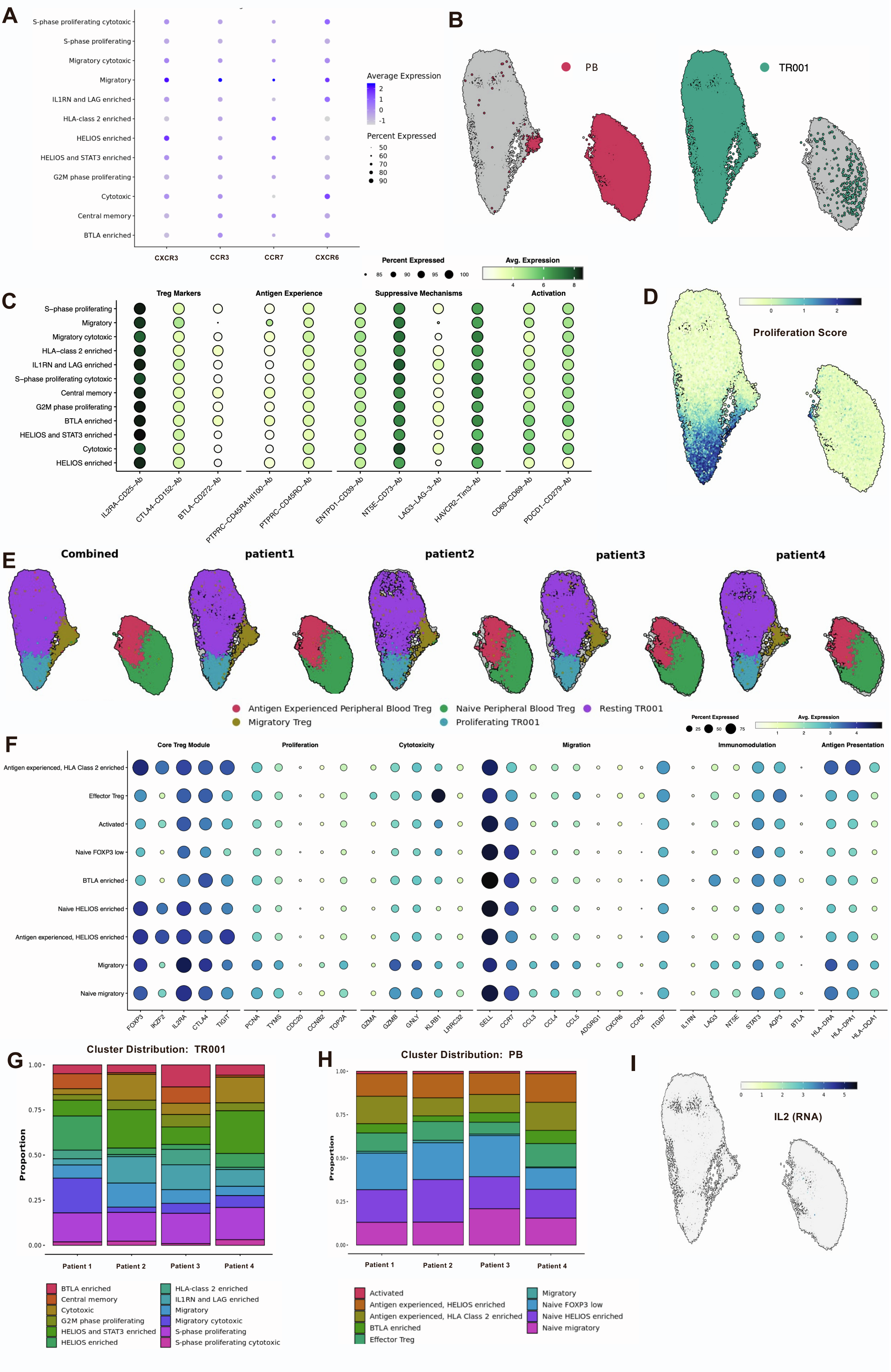
A
S-phase proliferating cytotoxic
S-phase proliferating
Migratory cytotoxic
Migratory
IL1RN and LAG enriched
HLA-class 2 enriched
HELIOS enriched
HELIOS and STAT3 enriched
G2M phase proliferating
Cytotoxic
Central memory
BTLA enriched
CXCR3
CCR3
CCR7
CXCR6
Average Expression
Percent Expressed
B
PB
TR001
C
Treg Markers
Antigen Experience
Suppressive Mechanisms
Activation
Percent Expressed
Avg. Expression
IL2RA–CD25–Ab
CTLA4–CD152–Ab
BTLA–CD272–Ab
PTPRC–CD45RA:HI100–Ab
PTPRC–CD45RO–Ab
ENTPD1–CD39–Ab
NT5E–CD73–Ab
LAG3–LAG–3–Ab
HAVCR2–Tim3–Ab
CD69–CD69–Ab
PDCD1–CD279–Ab
D
Proliferation Score
E
Combined
patient1
patient2
patient3
patient4
Antigen Experienced Peripheral Blood Treg
Naive Peripheral Blood Treg
Resting TR001
Migratory Treg
Proliferating TR001
F
Core Treg Module
Proliferation
Cytotoxicity
Migration
Immunomodulation
Antigen Presentation
Antigen experienced, HLA Class 2 enriched
Effector Treg
Activated
Naive FOXP3 low
BTLA enriched
Naive HELIOS enriched
Antigen experienced, HELIOS enriched
Migratory
Naive migratory
FOXP3 IKZF2 IL2RA CTLA4 TIGIT PCNA TYMS CDC20 CCNB2 TOP2A GZMA GZMB GNLY KLRB1 LRRC32 SELL CCR7 CCL3 CCL4 CCL5 ADGRG1 CXCR6 CCR2 ITGB7 IL1RN LAG3 NT5E STAT3 AQP3 BTLA HLA–DRA HLA–DPA1 HLA–DQA1
G
Cluster Distribution: TR001
Proportion
Patient 1
Patient 2
Patient 3
Patient 4
BTLA enriched
Central memory
Cytotoxic
G2M phase proliferating
HELIOS and STAT3 enriched
HELIOS enriched
HLA-class 2 enriched
IL1RN and LAG enriched
Migratory
Migratory cytotoxic
S-phase proliferating
S-phase proliferating cytotoxic
H
Cluster Distribution: PB
Activated
Antigen experienced, HELIOS enriched
Antigen experienced, HLA Class 2 enriched
BTLA enriched
Effector Treg
Migratory
Naive FOXP3 low
Naive HELIOS enriched
Naive migratory
I
IL2 (RNA)

Supplementary Figure 2, related to Figure 2: TR001 phenotyping by CITEseq.

(A) Average and percentage expression of key chemokines CXCR3, CCR3, CCR7, and CXCR6 across annotated TR001 Treg clusters. **(B)** UMAP of combined TR001 and peripheral Treg data grouped by sample type. **(C)** Bubble plot of average and percentage expression of key protein markers of Treg ontogeny, antigen experience, suppressive mechanisms and activation by annotated TR001 Treg clusters. **(D)** UMAP of combined TR001 and peripheral Treg data grouped by proliferation score (see methods). **(E)** Post integration (FastMNN) illustration of the cluster contribution by patient. **(F)** Bubble plot of average and percentage expression of key RNA markers of Treg ontogeny, proliferation, cytotoxicity, migration, suppressive mechanisms, and antigen presentation by annotated PB Treg cluster. **(G)** Distribution analysis showing the relative contribution of each TR001 cluster to the whole sample, split by patient. **(H)** Distribution analysis showing the relative contribution of each PB Treg cluster to the whole sample, split by patient. **(I)** UMAP of combined TR001 and peripheral Treg data illustrating the expression of IL2 transcripts across both sample types.

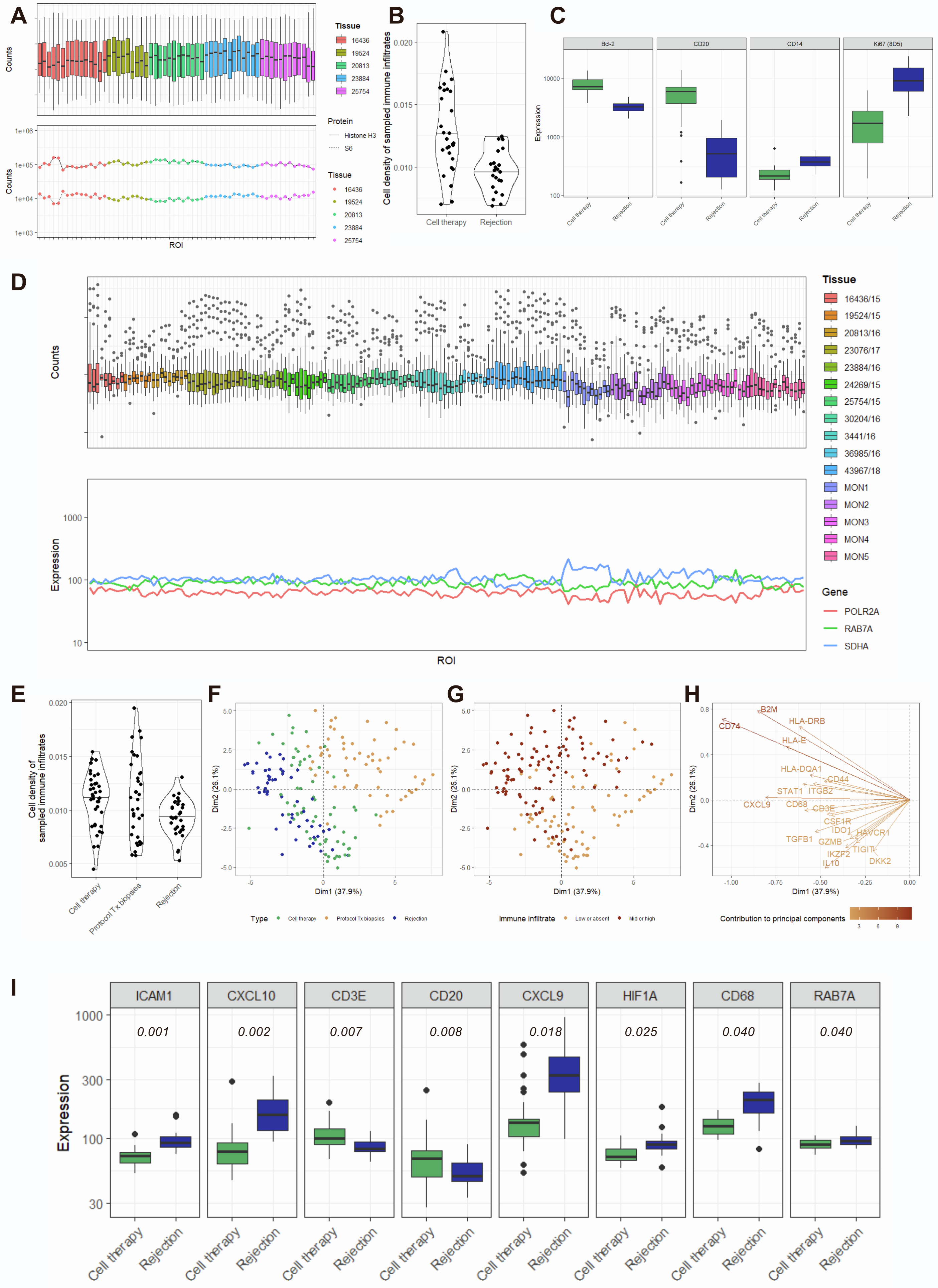
A
B
C
D
E
F
G
H
I
Tissue
16436
19524
20813
23884
25754
Protein
Histone H3
S6
Counts
ROI
Cell density of sampled immune infiltrates
Cell therapy
Rejection
Bcl-2
CD20
CD14
Ki67 (8D5)
Expression
16436/15
19524/15
20813/16
23076/17
23884/16
24269/15
25754/15
30204/16
3441/16
36985/16
43967/18
MON1
MON2
MON3
MON4
MON5
Gene
POLR2A
RAB7A
SDHA
Protocol Tx biopsies
Dim1 (37.9%)
Dim2 (26.1%)
Type
Immune infiltrate
Low or absent
Mid or high
Contribution to principal components
B2M
HLA-DRB
CD74
HLA-E
HLA-DQA1
CD44
STAT1
ITGB2
CXCL9
CD68
CD3E
CSF1R
IDO1
HAVCR1
TGFB1
GZMB
TIGIT
IKZF2
IL10
DKK2
ICAM1
CXCL10
CD3E
CD20
CXCL9
HIF1A
CD68
RAB7A
0.001
0.002
0.007
0.008
0.018
0.025
0.040
0.040

Supplementary Figure 3, related to Figure 3 and Figure 4: Quality control metrics for GeoMx Protein and RNA Assays

(A) Probe counts across each region of interest by tissue following histone H3 (top) or S6 (bottom) normalisation. **(B)** Box and whisker plots comparing the expression of Bcl-2, CD20, CD14, and Ki67 between cell therapy and rejection samples. **(C)** Violin plot illustrating the cell density of sampled immune infiltrates across cell therapy and rejection biopsies. **(D)** Probe counts across each region of interest following S6 normalisation (top) and expression of housekeeping genes across each region of interest following S6 normalisation (bottom). **(E)** Violin plot illustrating the cell density of sampled immune infiltrates across cell therapy, protocol, and rejection biopsies. **(F)** PCA plot illustrating the top two dimensions of variance grouped by sample type. **(G)** PCA plot illustrating the top two dimensions of variance grouped by degree of immune infiltrate. **(H)** PCA loading plot illustrating the contribution of the top 20 transcripts to the variance explained by the first and second dimensions. **(I)** Box and whisker plots comparing the expression of ICAM1, CXCL10, CD3E, CD20, CXCL9, HIF1α, CD68, and RAB78 between cell therapy and rejection samples (absolute unadjusted p values by Student's T test inset).

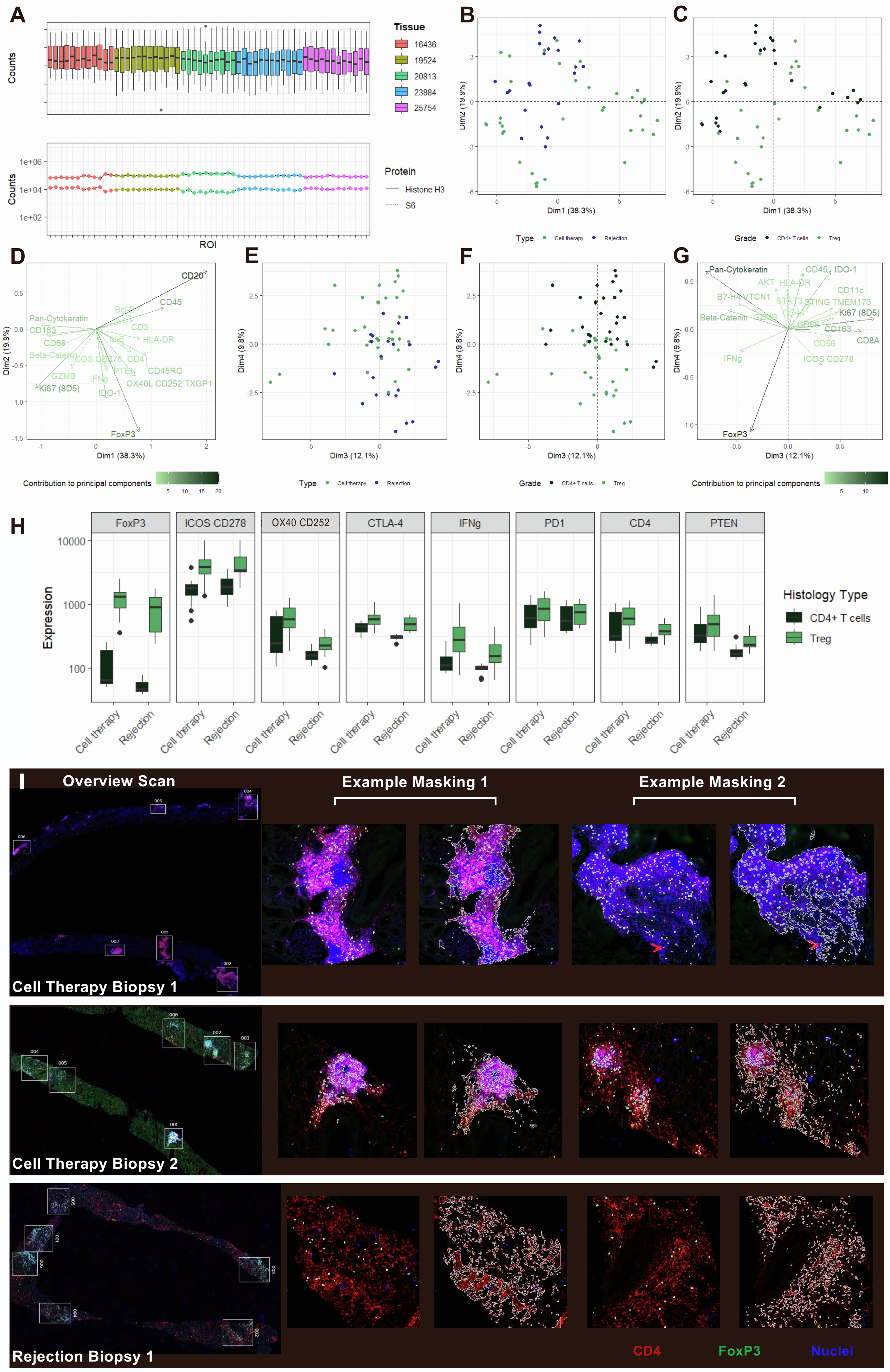

A
Counts
Tissue
16436
19524
20813
23884
25754
Counts
1e+06
1e+04
1e+02
Protein
Histone H3
S6
ROI
B
Dim2 (19.9%)
Dim1 (38.3%)
Type
Cell therapy
Rejection
C
Dim2 (19.9%)
Dim1 (38.3%)
Grade
CD4+ T cells
Treg
D
CD20
CD45
Bcl-2
Pan-Cytokeratin
CD163
CD3
HLA-DR
CD68
IL-6
Beta-Catenin
ICOS CD278
CD4
PTEN
CD45RO
GZMB
IFNg
OX40L CD252 TXGP1
Ki67 (8D5)
IDO-1
FoxP3
Dim2 (19.9%)
Dim1 (38.3%)
Contribution to principal components
E
Dim4 (9.8%)
Dim3 (12.1%)
Type
Cell therapy
Rejection
F
Dim4 (9.8%)
Dim3 (12.1%)
Grade
CD4+ T cells
Treg
G
Pan-Cytokeratin
CD45
IDO-1
AKT
HLA-DR
CD11c
B7-H4 VTCN1
STAT3
STING TMEM173
CD44
Ki67 (8D5)
Beta-Catenin
GZMB
CD68
CD163
CD8A
CD56
IFNg
ICOS CD278
FoxP3
Dim4 (9.8%)
Dim3 (12.1%)
Contribution to principal components
H
Expression
FoxP3
ICOS CD278
OX40 CD252
CTLA-4
IFNg
PD1
CD4
PTEN
Cell therapy
Rejection
Histology Type
CD4+ T cells
Treg
I
Overview Scan
Example Masking 1
Example Masking 2
Cell Therapy Biopsy 1
Cell Therapy Biopsy 2
Rejection Biopsy 1
CD4
FoxP3
Nuclei

Supplementary Figure 4, related to Figure 4: Quality control metrics for protein assay with rare cell masking

(A) Probe counts across each region of interest by tissue following histone H3 (top) or S6 (bottom) normalisation. **(B, C)** PCA plot illustrating the top two dimensions of variance grouped by sample type (B) and region type (C). **(D)** PCA loading plot illustrating the contribution of the top ranked protein markers to the variance explained by the first and second dimensions. **(E, F)** PCA plot illustrating the third and fourth dimensions of variance grouped by sample type (E) and region type (F). **(G)** PCA loading plot illustrating the contribution of the top ranked protein markers to the variance explained by the first and second dimensions (right). **(H)** Box and whisker plots comparing the expression of FOXP3, ICOS, OX40, CTLA4, IFNγ, PD1, CD4, and PTEN between cell therapy and rejection samples, grouped by region type. **(I)** Three colour immunofluorescence (Red: CD4, Green: FOXP3, Blue: DNA) illustrating selected regions from two cell therapy biopsies (top and middle panels) and one rejection biopsy (bottom panel). Regions are denoted by the white boxes. Zoomed in ROIs illustrating rare-cell collection masking strategy.

Control group

	C01	C02	C03	C04	C05	C06	C07	C08	C09	C10
Age at Tx	34	46	34	57	39	41	65	57	67	49
Ethnicity	White	White	White	White	White	White	White	White	White	White
Sex	Male	Male	Male	Female	Male	Male	Male	Male	Male	Male
Pre tx RRT	N	N	PD	N	HD	PD	N	PD	N	N
CMV	N	Y (9)	N	N	N	N	N	N	N	N
BKV	N	N	N	N	N	N	Y (4)	N	N	N
Rejection	N	N	N	N	N	N	N	Acute Banff 1a	N	N
Graft failure	N	Failure at 78 months	N	N	N	Failure at 96 months	N	N	N	N
Status	Alive	Dead	Alive	Alive	Alive	Alive	Alive	Alive	Alive	Alive

Cell therapy group

	Treg 01	Treg 02	Treg 03	Treg 04	Treg 05	Treg 06	Treg 07	Treg 08	Treg 09	Treg 10
Age at Tx	50	41	42	71	39	35	45	30	69	47
Ethnicity	White	White	Black	White	White	White	White	White	White	White
Sex	Male	Male	Female	Male	Male	Male	Male	Male	Male	Male
Pre tx RRT	N	HD	HD	N	N	N	N	N	N	N
CMV	N	N	N	N	Y (3)	N	N	N	N	N
BKV	N	N	N	Y (3)	Y (1)	N	N	N	N	N
Rejection	N	N	N	N	N	N	N	N	N	N
Graft failure	N	N	N	N	N	N	N	N	N	N
Status	Alive	Alive	Alive	Alive	Alive	Alive	Alive	Alive	Alive	Alive
MMF weaned	N	N	Y	Y	Y	N	Y	Y	N	Y
Tacrolimus monotherapy established	N	N	N	N	Y	N	Y	N	N	Y
Cell dose	1x10^6/kg	1x10^6/kg	1x10^6/kg	3x10^6/kg	6x10^6/kg	3x10^6/kg	6x10^6/kg	10x10^6.kg	10x10^6.kg	10x10^6.kg

Supplementary Table 1, related to Figure 1: Demographics of reference group and cell therapy treated patients. N = no. Y = yes. Y(N) = months post-transplant where event occurred. Tx = transplant. RRT = renal replacement therapy. PD = peritoneal dialysis. HD = haemodialysis.
